# Prospective comparison of preference and efficacy of adalimumab and infliximab for treating ulcerative colitis naive to antitumor necrosis factor therapy

**DOI:** 10.1097/MD.0000000000007800

**Published:** 2017-08-11

**Authors:** Tsutomu Mizoshita, Takahito Katano, Satoshi Tanida, Atsuyuki Hirano, Tomokatsu Miyaki, Keiji Ozeki, Yuka Suzuki, Naomi Sugimura, Hiromi Kataoka, Takashi Joh

**Affiliations:** a Department of Gastroenterology and Metabolism, Nagoya City University Graduate School of Medical Sciences; b Department of Gastroenterology, Nagoya City West Medical Center; c Department of Gastroenterology, Toyokawa City Hospital; d Department of Gastroenterology, Japanese Red Cross Nagoya Daini Hospital; e Department of Gastroenterology, Nagoya Memorial Hospital, Nagoya, Japan.

**Keywords:** adalimumab, infliximab, ulcerative colitis

## Abstract

There have been few reports on 2 tumor necrosis factor alpha inhibitors, infliximab and adalimumab, with respect to patient preference and efficacy in ulcerative colitis (UC).

We used questionnaires to evaluate the preference and reasons for drug choice between infliximab and adalimumab in UC patients naive to antitumor necrosis factor alpha therapy. We also analyzed the efficacy of infliximab and adalimumab prospectively and endoscopically before treatment and at 14 and 54 weeks.

Of the 25 UC patients, infliximab and adalimumab were chosen by 10 (40%) and 15 (60%), respectively. Patients who favored infliximab considered “fear of syringes” (7/10, 70%) as the most important influencing factor, whereas patients who favored adalimumab considered “ease of administration” (10/15, 66.7%) and “time required for therapy” (10/15, 66.7%) as the most important factors. There were no statistical differences in remission induction and maintenance between the infliximab and adalimumab groups with regard to response, remission, mucosal healing, steroid-free, and steroid-free remission rates at weeks 14 and 54.

The efficacy of adalimumab in remission induction and maintenance was equivalent to that of infliximab in UC patients naive to antitumor necrosis factor alpha therapy in this prospective study, but more patients preferred adalimumab.

## 1 Introduction

Inhibition of tumor necrosis factor alpha (TNF-α) is very important for the control of inflammatory lesions in ulcerative colitis (UC). Two TNF-α inhibitors, infliximab (IFX, a chimeric monoclonal antibody) and adalimumab (ADA, a fully human monoclonal antibody), are approved for the treatment of UC in Japan. The primary difference between these 2 TNF-α inhibitors is the mode (intravenous vs subcutaneous) and interval (2 months vs 2 weeks) of administration.^[1]^ In the CHOOSE TNF TRIAL, a systematic assessment of factors influencing the preferences of Crohn disease (CD) patients in selecting an anti-TNF-α agent that was conducted in Switzerland, the majority of patients preferred anti-TNF-α medications that were administered by subcutaneous injection rather than intravenous infusion, suggesting that ease of use and time required for therapy were 2 major influencing factors.^[2]^ Racial difference may also influence the selection of TNF-α inhibitors, because the East Asian culture is likely to be interdependent, whereas the western culture is more independent.^[1,3]^ To the best of our knowledge, there are no studies on whether patients with UC prefer intravenous IFX or subcutaneous ADA. Therefore, we believe that it is of interest to determine the anti-TNF medication of choice in this patient population in Japan, and to evaluate the underlying reasons for this choice. Patients with inflammatory bowel disease (IBD), including UC and CD, have a quality of life that is adversely affected. In a survey by the European Federation of Crohn's and Ulcerative Colitis Associations, 75% of patients reported symptoms affecting their ability to enjoy leisure activities, and two-thirds felt that their work performance was affected.^[4]^

Regarding comparisons of the efficacy of IFX and ADA, there have been no head-to-head comparison trials in UC patients.^[5]^ An indirect comparison meta-analysis showed that IFX and ADA were comparable in efficacy at 52 weeks of maintenance treatment, whereas IFX was statistically more effective than adalimumab in the induction of remission in moderate-to-severe UC patients naive to anti-TNF-α therapy.^[6]^ However, a network meta-analysis and prospective study of a single-center cohort showed no difference in outcomes between IFX and ADA in patients with UC.^[7–9]^ To date, there have been no prospective head-to-head comparison trials of the efficacy between IFX and ADA in East Asian patients with UC naive to anti-TNF-α therapy using endoscopic analyses.

Therefore, in this study, we used questionnaires to evaluate the preference and reasons contributing to the choice of IFX or ADA in UC patients naive to anti-TNF-α therapy. We also analyzed the efficacy of IFX and ADA prospectively, using the Mayo score and endoscopic activity index (EAI) as a measure of disease activity before treatment and at weeks 14 and 54.

## 2 Patients and methods

### 2.1 Study design

A prospective, questionnaire-based, and clinical survey (NCU-UC-CHOOSE TRIAL), approved by the Institutional Review Board at Nagoya City University Hospital, was performed in 25 UC patients who had never taken either of the 2 standard anti-TNF medications (IFX and ADA). All of the patients were naive to anti-TNF-α therapy and resistant to or untreatable with conventional therapy, and they participated in this trial between August 2013 and April 2016. Disease activity before and after anti-TNF-α therapy was measured using the Mayo score [also known as the disease activity index (DAI)] and the EAI.^[10,11]^ Patients were included who had a DAI ≥ 6 points and an EAI ≥ 2 points before the start of anti-TNF therapy. The exclusion criteria were as follows: age less than 20 years, severe infection, severe heart and renal disorders, pregnant or nursing, malignancy, and previous colectomy. Before the start of IFX and ADA, the bacterial infectious enteritis was ruled out by stool cultures. *Clostridium difficile* infection was ruled out by *C. difficile* toxin testing and stool cultures. Cytomegalovirus infection was ruled out by pathological analysis of lesions.^[11]^ After reading a brief description of IFX and ADA, all of the eligible participants were provided a questionnaire to determine their treatment preference after informed consent was obtained.^[1]^ According to the protocol, IFX was administered at 5 mg/kg to patients with active UC at weeks 0, 2, and 6, and intravenous IFX injections of 5 mg/kg were administered as maintenance doses every 8 weeks thereafter.^[12]^ ADA was administered subcutaneously at 160 mg at week 0, and 80 mg at week 2, and subsequent subcutaneous doses of 40 mg were given as maintenance doses every other week thereafter, according to the protocol.^[13]^ Endoscopy was conducted within 1 week before the start of IFX or ADA, and the second and third endoscopic observations were performed to evaluate mucosal healing at 14 of 54 weeks after the patient was started on anti-TNF-α therapy, using the EAI and Mayo score.^[11]^ The response, remission, mucosal healing, steroid-free, and steroid-free remission rates at weeks 14 and 54 were evaluated as previously described.^[13]^ Efficacy endpoints analyzed included response per full Mayo score [decrease of ≥3 points and ≥30% from baseline along with a decrease in the rectal bleeding subscore (RBS) ≥ 1 or an absolute RBS of ≤1], remission (full Mayo score ≤2 with no individual subscore ≥1), and mucosal healing (endoscopy subscore ≤1) at weeks 14 and 54.^[13]^ The patient was considered to have treatment failure as the drop out, when any assessment score was noted to worsen or remain unchanged with the aggravation of the physician's global assessment, suggesting that the continuance of the anti-TNF therapy was not appropriate. In the patients taking at systemic corticosteroids at baseline, the cases having the stop of corticosteroids were defined as steroid-free during the anti-TNF therapy. The patients having both remission and the stop of corticosteroids were defined as the steroid-free remission.

### 2.2 Questionnaires

The patients were asked to participate in the study during their regular outpatient visits or during hospital stays.^[2]^ The patients were first asked to read a 1-page description of 2 anti-TNF drugs (IFX and ADA) that are currently available in Japan. The description of the drugs included the mode; time, place, and interval of administration; approval date in Japan; efficacy; cost; and adverse effects.^[1]^ After reading the description, patients were asked to answer the questionnaire with the following questions:

“May we use the result of your questionnaire in this study?”

“Which of the anti-TNF drugs would you choose based on information provided in this study, IFX or ADA?”

“What factor influenced your choice of anti-TNF medication most (multiple answers possible)? For example, administration route (intravenous IFX vs. subcutaneous ADA), administration time, time intervals between doses (2 months vs. 2 weeks), duration approved for use (2010 vs. 2013 in Japan), and adverse events.”

If there were multiple answers, the most important factor for choosing an anti-TNF drug was determined by the participants themselves.

### 2.3 Statistical analyses

The differences between IFX and ADA with regard to the factors that influenced drug choice were assessed using the Fisher exact test. *P* values <.05 were considered statistically significant.

## 3 Results

### 3.1 Patient selection of a specific anti-TNF medication, and patient characteristics

The baseline characteristics of the 25 biologically naive patients with UC who selected IFX (n = 10) or ADA (n = 15) are summarized in Table 1. The patients’ selections of a specific anti-TNF drug are summarized in Table 2. IFX and ADA were chosen by 10 (40%) and 15 (60%) patients, respectively. The patients who favored IFX considered “fear of syringes” (7/10, 70%) as the most important influencing factor, followed by “scientific evidence for efficacy” (4/10, 40%) and “duration approved for use” (3/10, 30%). Patients who favored ADA considered “ease of administration” (10/15, 66.7%) and “time required for therapy” (10/15, 66.7%) as the most important factors, followed by “time intervals between medication” (7/15, 46.7%) and “duration approved for use” (2/15, 13.3%).

**Table 1:**
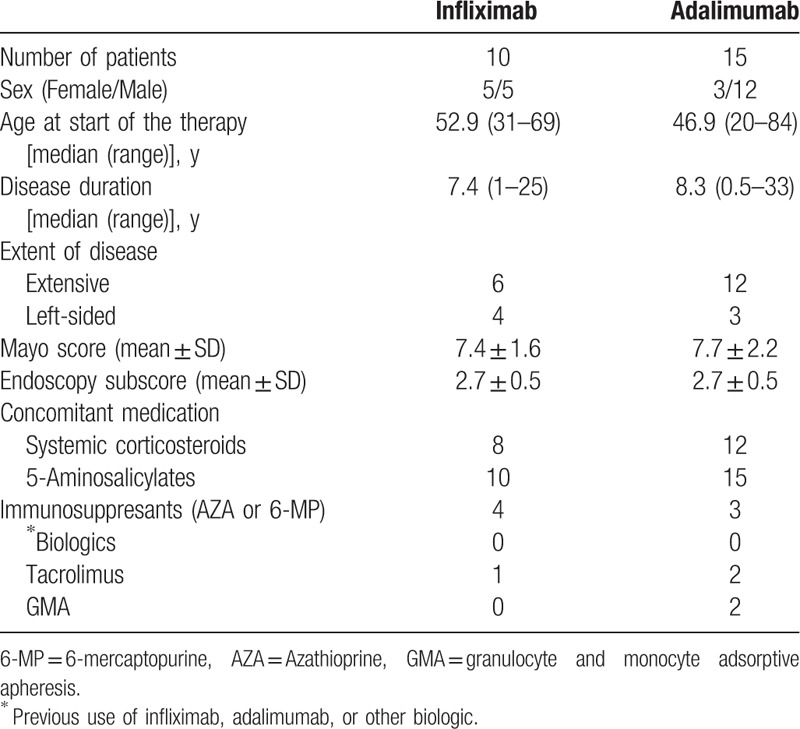
Baseline characteristics of biologically naive patients with moderate-to-severe ulcerative colitis.

**Table 2:**
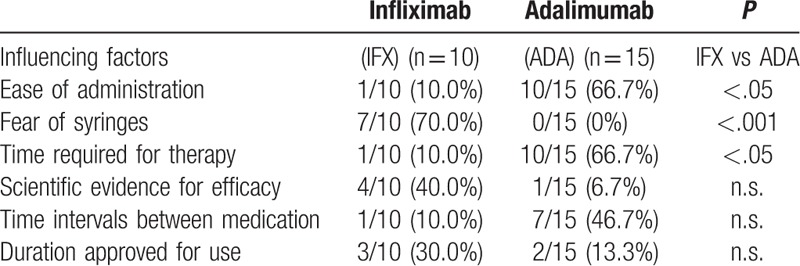
Factors influencing patient's choice of a specific anti-TNF-α drug, stratified by drug.

### 3.2 Response, remission, mucosal healing, steroid-free, and steroid-free remission rates at week 14

The response, remission, mucosal healing, steroid-free, and steroid-free remission rates at week 14 are summarized in Table 3. No patients had treatment failure of anti-TNF drug therapy at week 14. The response, remission, mucosal healing, steroid-free, and steroid-free remission rates at week 14 were 7 of 10 (70%), 4 of 10 (40%), 5 of 10 (50%), 4 of 8 (50%), and 2 of 8 (25%), respectively, in the IFX group and were 11 of 15 (73.3%), 6 of 15 (40%), 7 of 15 (46.7%), 8 of 12 (66.7%), and 4 of 12 (33.3%), respectively, in the ADA group. There were no statistical differences between IFX and ADA groups for each factor at week 14 (Table 3).

**Table 3:**
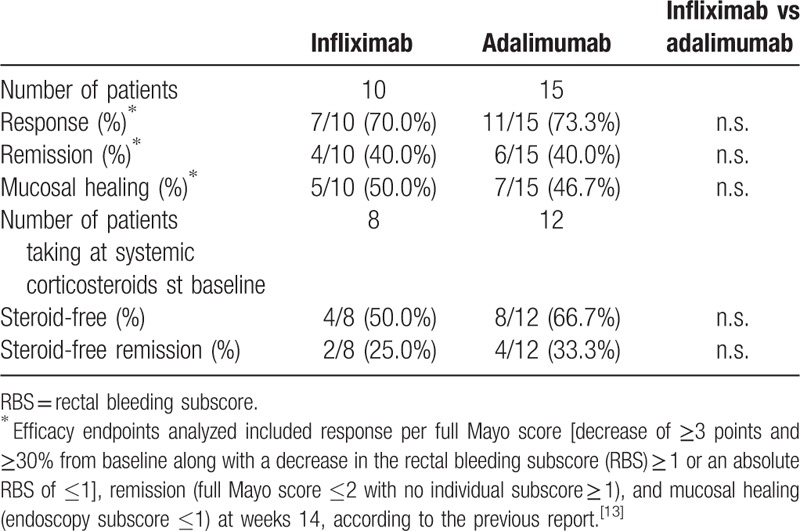
Response, remission, mucosal healing, steroid-free, and steroid-free remission rates at week 14 in biologically naive patients with ulcerative colitis treated with infliximab or adalimumab.

### 3.3 Response, remission, mucosal healing, steroid-free, and steroid-free remission rates at week 54

The response, remission, mucosal healing, steroid-free, and steroid-free remission rates at week 54 are summarized in Table 4. The response, remission, mucosal healing, steroid-free, and steroid-free remission rates at week 52 were 7 of 10 (70%), 6 of 10 (60%), 6 of 10 (60%), 7 of 8 (87.5%), and 5 of 8 (62.5%), respectively, in the IFX group and were 10 of 15 (66.7%), 8 of 15 (53.3%), 8 of 15 (53.3%), 7 of 12 (58.3%), and 5 of 12 (41.7%), respectively, in the ADA group. There were also no statistical differences between IFX and ADA groups for each factor at week 54 (Table 3). In the IFX group, 2 patients had treatment failure before week 54, both of whom received additional tacrolimus therapy. In the ADA group, 3 patients had treatment failure before week 54. One patient received additional tacrolimus therapy, one patient switched from ADA to IFX after additional azathioprine therapy, and the remaining patient received additional azathioprine therapy after being given an increased dose of prednisolone.

**Table 4:**
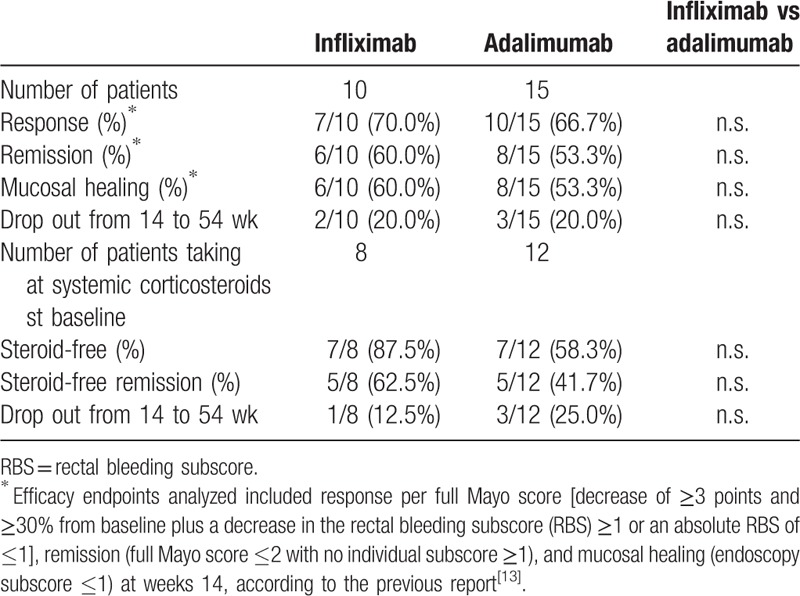
Response, remission, mucosal healing, steroid-free, and steroid-free remission rates at week 54 in biologically naive patients with ulcerative colitis treated with infliximab or adalimumab.

## 4 Discussion

Regarding the choice of TNF-α inhibitor, this is the first prospective real-world study in Japan to show that UC patients naive to anti-TNF-α therapy preferred ADA to IFX. The patients who favored IFX considered “fear of syringes” (7/10, 70%) as the most important influencing factor, while patients who favored ADA considered “ease of administration” (10/15, 66.7%) and “time required for therapy” (10/15, 66.7%) as the most important factors. The CHOOSE TNF TRIAL showed that CD patients preferred subcutaneous injection to intravenous infusion, suggesting that ease of use and time required for therapy were 2 major factors influencing their selection of a specific TNF-α inhibitor.^[2]^ However, the CHOICE Study demonstrated that a large number of anti-TNF-naive Korean patients with CD preferred the intravenous infusion of TNF-α inhibitor, suggesting that the reassuring effect of a doctor's presence might have been the main contributing factor for this decision.^[1]^ Racial differences may also affect the selection of TNF-α inhibitor, as the East Asian culture is likely to be interdependent, whereas the western culture is more independent.^[1,3]^ In patients with IBD, there was a trend toward patient preference for intravenous IFX treatment compared with subcutaneous ADA, suggesting that the difference may be due to the frequency of administration, mode of administration, or differing “times in the market-place,” as IFX had been approved for a longer period of time in CD.^[14]^ In patients with chronic inflammatory conditions (e.g., rheumatoid arthritis, plaque psoriasis, psoriatic arthritis), the subcutaneous routes of anti-TNF-α medications were preferred to intravenous routes patients in the entire population, but intravenous routes were preferred to subcutaneous routes in CD patients.^[15]^ Patients with rheumatoid arthritis may be immobile, leading to difficulty going to the hospital. Thus, they may be happier self-administering medication compared with IBD patients.^[14]^ In the present study, the costs of 2 anti-TNF-α medications were not important for the patients with UC with the regard to the choice of IFX and ADA. We consider the reason that UC is the disease of the publicly funded health care in Japan, and there are no differences of the costs between IFX and ADA. Further studies in a large population should be performed to clarify which TNF-α inhibitor is preferred by UC patients, as our present prospective trial was in a small population with the tendency that the median age of the cohort is rather high.

To the best of our knowledge, this is the first study to show that there were no differences between IFX and ADA with regard to response, remission, mucosal healing, steroid-free, and steroid-free remission rates at weeks 14 and 54 in Japanese UC patients naive to anti-TNF-α therapy. This is also the first head-to-head comparison trial of the efficacy of remission induction and maintenance between IFX and ADA in this patient population.^[5]^ An indirect comparison meta-analysis showed that IFX is statistically more effective than ADA in the induction of remission in patients with moderate-to-severe UC naive to anti-TNF-α therapy, whereas there was no difference between IFX and ADA regarding efficacy of maintenance treatment at week 52.^[6]^ In our prospective study, however, the efficacy of ADA in remission induction was equivalent to that of IFX at week 14 in UC patients naive to anti-TNF-α therapy. Similarly, no difference in outcomes was identified between IFX and ADA in UC.^[7]^ A network meta-analysis demonstrated that no single agent is clinically superior to the others; thus, other factors such as cost, safety, route of administration, and patient preference should dictate the choice of anti-TNF agents, suggesting the importance of a randomized prospective comparative efficacy trial between IFX and ADA in UC.^[8]^ A prospective study of a single-center cohort demonstrated that both IFX and ADA were effective in generating induction and maintenance responses in UC patients upon evaluation of clinical features, although no endoscopic findings were analyzed.^[9]^ Taking into account the above-mentioned previous reports and our present data, we consider that the efficacy of ADA in remission induction and maintenance is equivalent to that of IFX in UC patients naive to anti-TNF-α therapy. However, additional studies in a larger population are needed to confirm these results, considering that the drop-out patients (n = 5) from the study was rather high.

Regarding the efficacy of IFX therapy in UC patients naive to anti-TNF-α therapy, the rates of clinical response at weeks 8, 30, and 54 were 54.8%∼69.4%, 46.2%∼52.1%, and 45.5%.^[12,16]^ The rates of clinical remission at weeks 8, 30, and 54 were 20.2%∼38.8%, 21.2%∼33.9%, and 34.7%.^[12,16]^ The rates of mucosal healing at weeks 8 and 30 were 46.2% and 41.3%.^[12]^ In the present prospective real-world study of IFX in Japan, the rates of response, remission, mucosal healing, steroid-free, and steroid-free remission rates were 70.0%, 40.0%, 50.0%, 50.0%, and 25.0%, respectively, at week 14, and were 70.0%, 60.0%, 60.0%, 87.5%, and 62.5%, respectively, at week 54. In the ADA groups of anti-TNF naive patients with UC, the rates of clinical response at weeks 8 and 52 were 50% and 31%∼53.6%.^[13,17]^ The rates of clinical remission at weeks 8 and 52 were 18.5%∼21.3% and 22%∼65%.^[13,17–20]^ The rates of mucosal healing at weeks 8 and 52 were 44% and 29%∼50%.^[13,17,20]^ In our prospective real-world study of ADA in Japan, the rates of response, remission, mucosal healing, steroid-free, and steroid-free remission rates were 73.3%, 40.0%, 46.7%, 66.7%, and 33.3%, respectively, at week 14, and were 66.7%, 53.3%, 53.3%, 58.3%, and 41.7%, respectively, at week 54. Thus, we consider that both IFX and ADA have good efficacy in UC.

In conclusion, the efficacy of ADA in remission induction and maintenance was equivalent to that of IFX in UC patients naive to anti- TNF-α therapy in this prospective study, but most patients preferred ADA.
